# Efficacy and safety of anterior cervical discectomy and fusion (ACDF) through mini-incision and posterior laminoplasty (LAMP) for treatment of long-level cervical spondylosis: a retrospective cohort study

**DOI:** 10.1186/s12893-022-01567-2

**Published:** 2022-03-25

**Authors:** Yingkai Zhang, Guangling Yang, Tianyao Zhou, Yanchao Chen, Zhenchao Gao, Weili Zhou, Yutong Gu

**Affiliations:** 1 Department of Orthopaedic Surgery, Zhongshan Hospital Fudan University, Fenglin Road 180, Shanghai City, 200032 People’s Republic of China; 2 Department of Orthopaedic Surgery, Jinshan Hospital of Fudan University, Shanghai City, 201508 People’s Republic of China; 3 Department of Orthopaedic Surgery, Shanghai Public Health Clinical Center, Fudan University, Shanghai City, 201508 People’s Republic of China; 4 Shanghai Southwest Spine Surgery Center, Shanghai City, 201508 People’s Republic of China

**Keywords:** ACDF, LAMP, Long-level cervical spondylosis, Mini-incision

## Abstract

**Background:**

The efficacy and safety of anterior cervical discectomy and fusion (ACDF) through mini-incision and posterior laminoplasty for long-level cervical spondylosis were investigated.

**Method:**

From January 2018 to September 2019, clinical patients data with 3–4 segments (C3–7) cervical spondylotic radiculopathy, cervical spondylotic myelopathy, or mixed cervical spondylosis who received ACDF (42 cases) throughwith mini-incision or LAMP (36 cases) treatment were retrospectively collected and analyzed. The operative time, bleeding volume, incisive length, and hospital stay were recorded. Moreover, the intervertebral height, functional segment height, cervical lordosis, cervical hyperextension and hyperflexion range-of-motion (ROM) and ROM in all directions of the cervical spine before and after the operation were measured. Additionally, all relevant postoperative complications were also recorded. Then, the therapeutic effects of both surgical methods were investigated.

**Results:**

Patients in the ACDF group had less bleeding, shorter incision, and fewer hospitalization days than the LAMP group. There was no significant difference in JOA, VAS score of the upper limb, NDI score after surgery between two groups. Postoperative intervertebral height and functional segment height in the ACDF group were significantly higher than those before the operation, and postoperative functional segment height of the ACDF group was significantly higher than that of the LAMP group. Moreover, the postoperative cervical lordosis angle in the ACDF group was significantly larger than the LAMP group. There was no significant difference between preoperative and postoperative ROM in all directions of the cervical spine for the two groups.

**Conclusions:**

Both ACDF through mini-incision and LAMP are effective treatments for long-level cervical spondylosis. However, ACDF through mini-incision shows minor trauma, less bleeding, fast recovery, and it is beneficial for cervical lordosis reconstruction.

## Introduction

Cervical spondylosis (CS) is the most common nerve root and spinal cord degenerative disorder in adults whose diagnosis includes symptoms, signs, and various imaging examinations [1]. The treatment for CS includes anterior and posterior surgery [2]. In 1958, anterior cervical discectomy and fusion (ACDF) were first proposed to treat CS [3]. Since then, the safety and effectiveness of short-segment ACDF to alleviate cervical nerve symptoms have been verified by many studies; however, long-stage (> 3 segments) ACDF was seldomly reported [4, 5]. Additionally, it has been reported that long-segment surgery might induce several complications such as dysphagia [6, 7]. With the development of technology, a novel zero-profile implant was used for ACDF [8]. Thus, it is possible to have a shorter incision and less damage to the esophagus in the long-level ACDF. In 1981, Hirabayashi et al. [9] first performed posterior laminoplasty (LAMP) to treat the ossification of the posterior longitudinal ligament (OPLL). Although the pathological factors leading to compression had not been treated, the pressure on the nerve could be indirectly alleviated as the nerve drifted backward like a bowstring following decompression. Nevertheless, several patients who received long-segment LAMP showed C5 paralysis, reduction of cervical range-of-motion (ROM), axial cervical pain, and other complications [10]. Due to poor daily habits combined with the aging of the general population, the incidence of long-level CS has increased. However, there remains controversy regarding the surgical treatment of long-level CS in the absence of severe OPLL [11–13]. This study aims to investigate the clinical efficacy of ACDF through mini-incision (using ROI-C plug-in interbody fusion cage) and LAMP in long-level CS treatment. These findings will provide greater insight to clinicians and can be used to improve patient outcomes.

## Materials and methods

This study was approved by the ethics committee of Zhongshan Hospital Fudan University and informed consent was obtained from all patients.

From January 2018 to September 2019, clinical data were retrospectively collected from patients with long-level (3–4 segments) CS who received ACDF (42 cases) or LAMP (36 cases) treatment. How to choose anterior or posterior approach was based on the contraindications for ACDF and LAMP. Sometimes, it also depended on habits and technology of surgeon and individual differences in patients.

Inclusive criteria: (1) corresponding symptoms of spinal cord or nerve root compression; (2) 3–4 segment (C3–7) disc herniation on MRI and CT; (3) conservative treatment is not effective; (4) complete image data; (5) the follow-up time > 24 months;

Contraindications for ACDF: (1) developmental cervical spinal stenosis; (2) total loss of disc space; (3) OPLL; (4) ossification of the ligamentum flavum or main compressive factors located at the dorsal of the spinal cord.

Contraindications for LAMP: (1) cervical instability; (2) cervical kyphosis of > 10°or cases with negative modified K-line on magnetic resonance imaging.

Exclusive criteria: (1) cervical tumor, trauma, or infection; (2) previous spine surgery; (3) lack of complete imaging data or follow-up time < 24 months.

### Surgical procedures

**Fig. 1:**
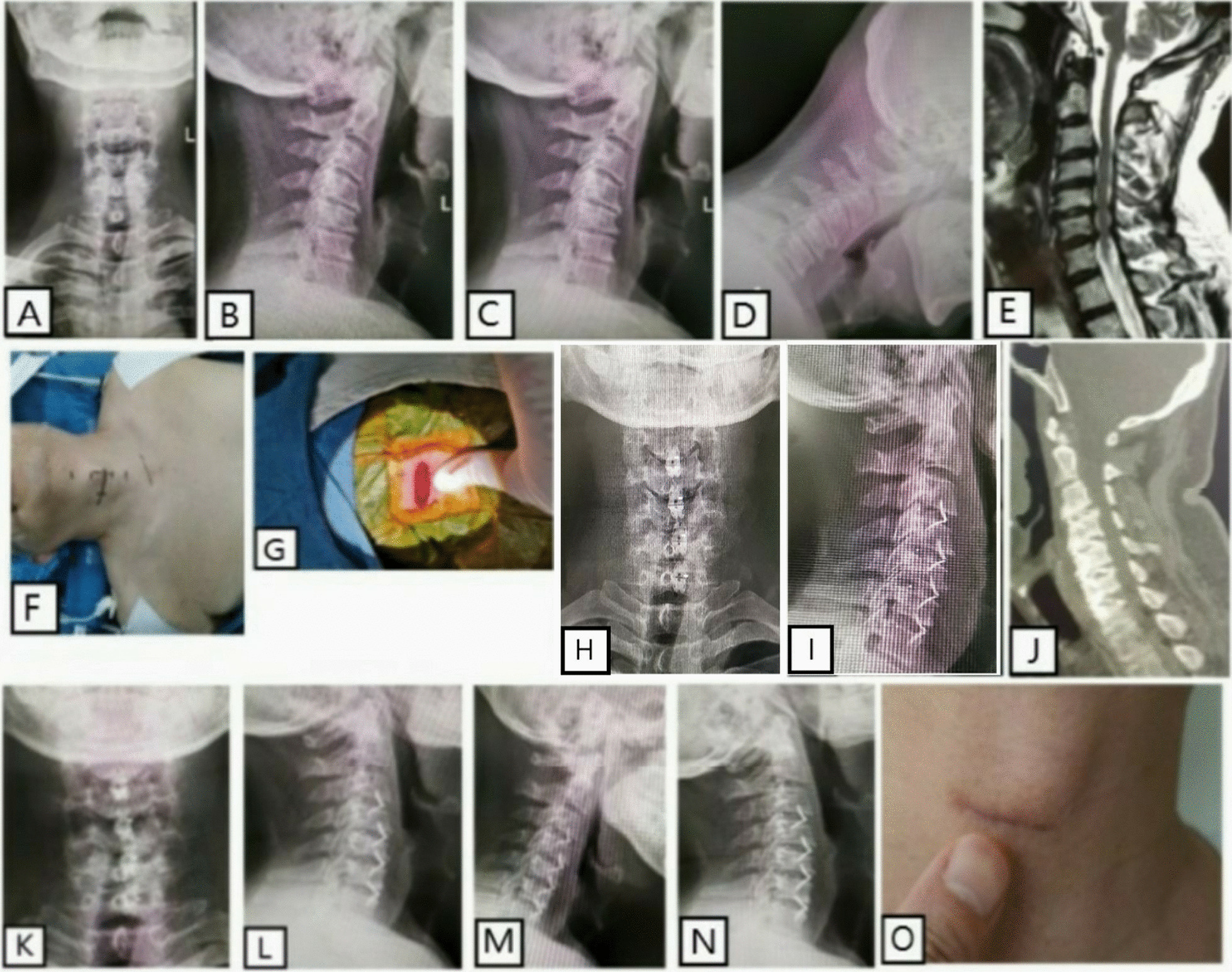
ACDF treatment for multisegments CS. **A** preoperative anteroposterior radiograph. **B** Preoperative lateral radiograph. **C**–**D** Preoperative hyperextension and flexion radiograph. **E** Preoperative cervical MR. **F** Preoperative incision. **G** Intraoperative incision. **H** Postoperative anteroposterior radiograph. **I** Postoperative lateral radiograph. **J**–**L** Postoperative CT and X-ray at the follow-up. **M**–**N** Postoperative hyperextension and flexion radiograph, and **O** postoperative incision

Before ACDF, patients underwent general anesthesia and were placed in the neck hyperextension supine position. A 2–3 cm right anterior transverse incision was made approximately parallel to the lower edge of the thyroid cartilage. Because ROI-C plug-in interbody fusion cage (Zimmer Holdings, Inc. America) was instead of long anterior cervical plate in ACDF, the incision was shorter than traditional ACDF. The skin, subcutaneous superficial fascia, platysma, and deep fascia were then cut in turn. The approach extended between the cervical visceral and vascular sheath to expose the diseased intervertebral space located at the most cephalic location from C3–7. Vertebral nails were then drilled into the head and tail vertebral bodies, respectively. The tail vertebral body nail deviated to the lower endplate. After partial discectomy, a Caspar distractor was used to open the diseased intervertebral space. The nucleus pulposus was removed, the cartilage of the endplate was scraped, and the osteophyte at the posterior edge of the vertebral body was removed. The posterior longitudinal ligament was then opened to expose the dual sac. The bilateral intervertebral foramen was then enlarged to complete the neurologic decompression. After the model test, a suitable ROI-C plug-in interbody fusion cage (Zimmer Holdings, Inc. America) filled with allograft bone was inserted into the intervertebral space. Once the position of the cage was satisfactory, as assessed by fluoroscopy, the self-locking plug-in was inserted. The intervertebral space of other lesions was treated using the same method. Once completed, each layer was closed with a suture and a negative pressure drainage tube was placed (Fig. 1). After the operation, neck immobilization was maintained for 4 weeks (Fig. 1).

**Fig. 2:**
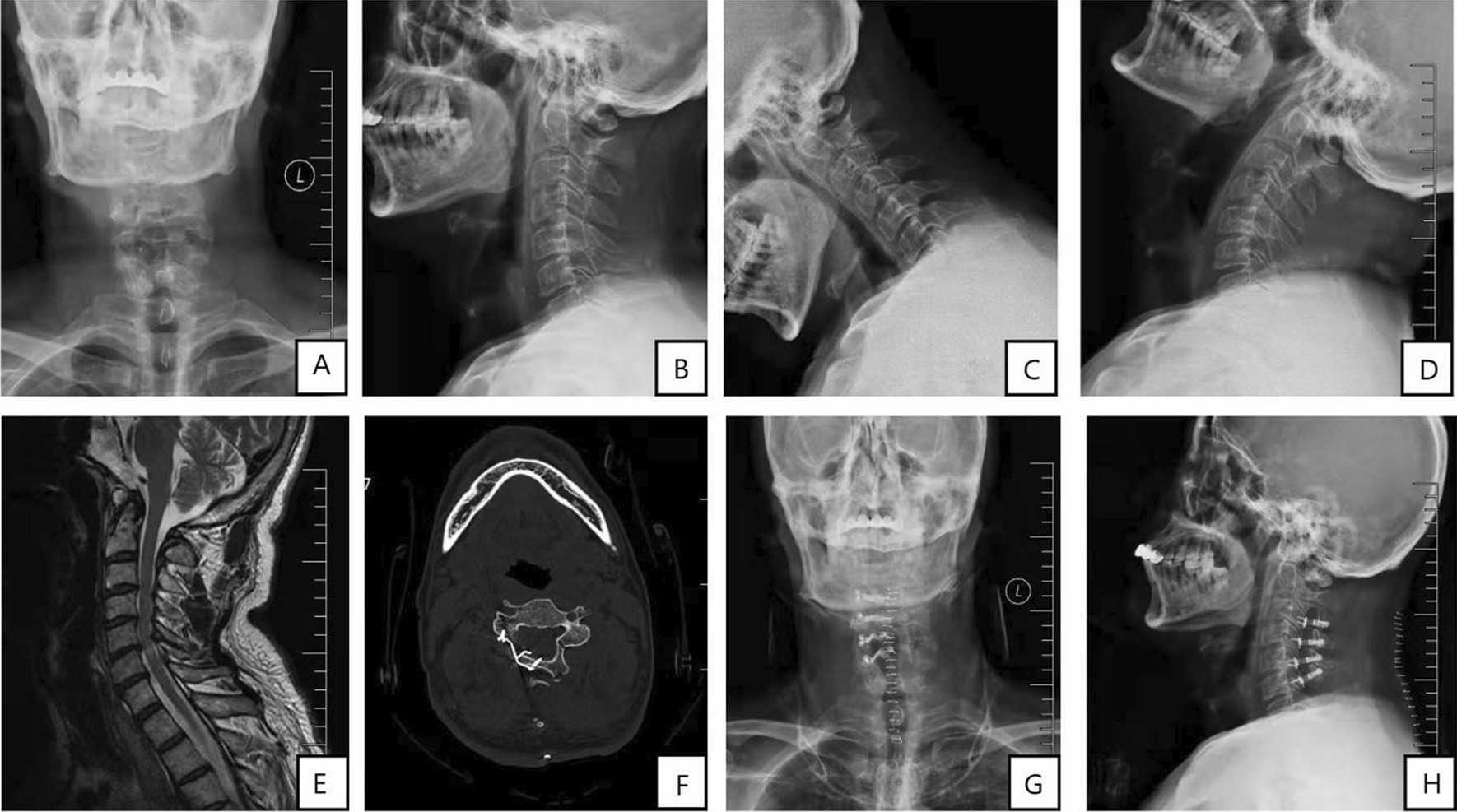
LAMP treatment for multisegments CS. **A** Preoperative anteroposterior radiograph. **B** Preoperative lateral radiograph. **C**–**D** Preoperative hyperextension and flexion radiograph. **E** Preoperative cervical MR. **F** Postoperative CT. **G** Postoperative anteroposterior radiograph; and **H** Postoperative lateral radiograph

LAMP was performed as previously described [14]. Briefly, the paravertebral muscles were detached from both the right and left sides of the spinous processes. The laminae were split between the laminae and facets at the unilateral side with severe neurologic compression and, using a high-speed air-burr drill, gutters were made between the laminae and facets at the contralateral side which acts as hinges. The laminae and spinous processes were turned over and small plates (Weigao Holdings, Inc. China) were fixed between the laminae and facets at the open side. Neurologic decompression was performed for 3 or 4 levels from C3–7. Each layer was closed with a suture and a negative pressure drainage tube was placed. Patients were instructed to wear a neck collar for 3–4 weeks (Fig. 2).

When the drainage volume < 20 ml/24 h remove the drainage tube and Patients can begin to walk. Two experienced spine surgeon performed the operations mentioned above.

### Data recording

#### The operation-related data

Operation time, operation bleeding, incision length, and average hospital stay were collected for subsequent analysis.

#### Clinical follow-up data

**Fig. 3:**
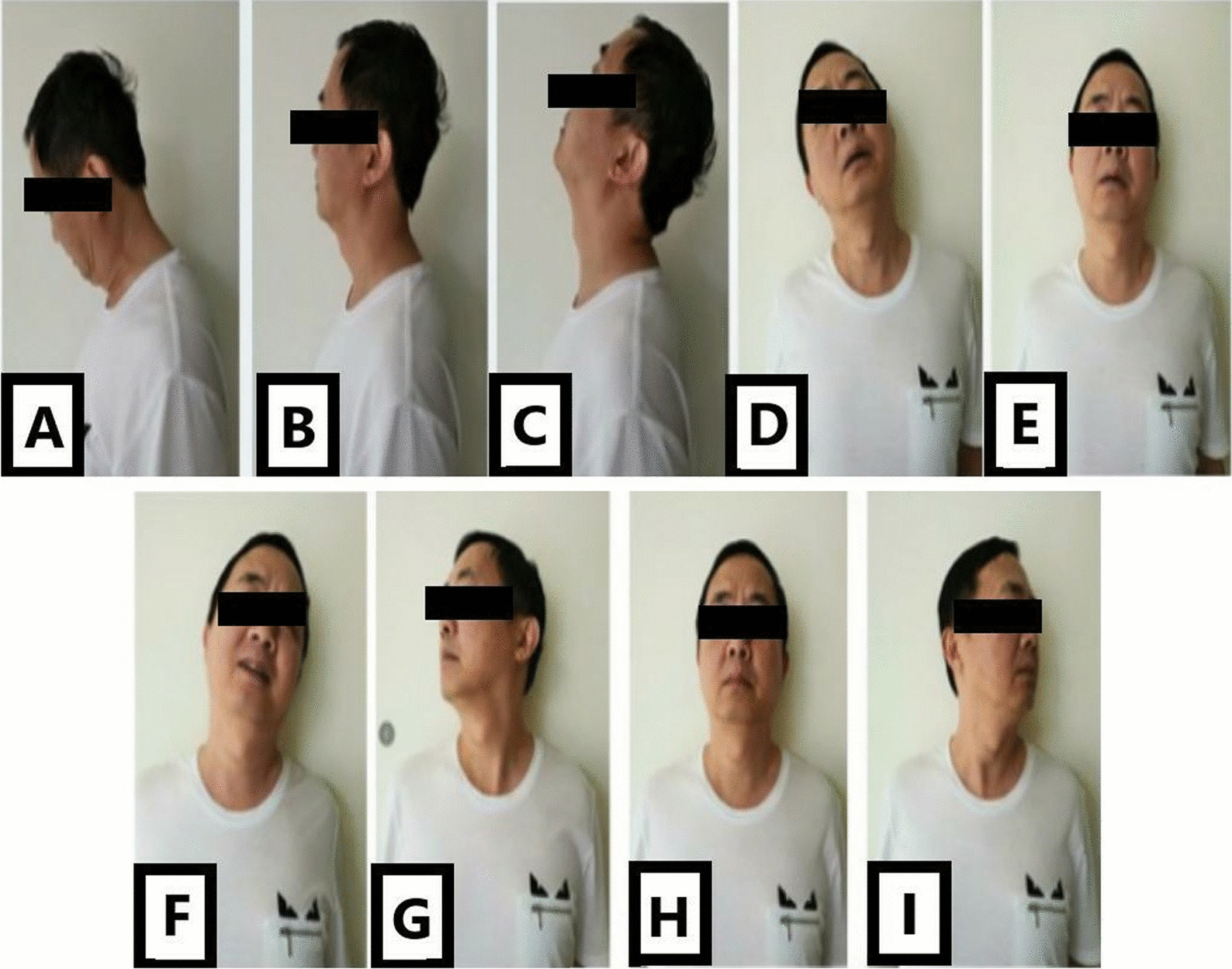
Measurement of cervical flexion and extension, lateral flexion, and axial rotation angle at postoperative follow-up. **A** Cervical hyperflexion position. **B** Neutral lateral position of the cervical spine. **C** Cervical hyperextension position. **D** Right cervical flexion position. **E** Neutral position of the cervical spine. **F** Left cervical flexion position. **G** Right axial rotation position of the cervical spine. **H** Neutral position of the cervical spine, and **I** Left axial rotation position of the cervical spine

The JOA score, JOA score improvement rate, and NDI score, VAS of the upper limb before the operation and 24 months after the operation were recorded. Neurologic function and cervical spine function before and after the operation were evaluated. ROM in all directions of the cervical spine before and 24 months after the operation was measured, including flexion, extension, lateral flexion, and rotation, and the loss rate of ROM was calculated (Fig. 3) [15]. The subjective satisfaction of the patients regarding clinical efficacy at 2-year follow-up was evaluated based on Odom criteria [16].

### Imaging data

The anterioposterior and lateral X-ray, as well as hyperextension and hyperflexion lateral X-ray of the cervical spine, 3-D CT were taken before, immediately after the operation, as well as at 3, 6, 12, and 24 months after the operation. The preoperative and postoperative intervertebral height, functional (surgical) segment height, cervical lordosis angle (C2–7 Cobb angle), cervical hyperextension and hyperflexion ROM (cervical lordosis angle difference was based on hyperextension and hyperflexion lateral film) were measured and whether there was subsidence of the fusion cage after the operation (loss of intervertebral height greater than 2 mm was defined as subsidence) was identified. The classification standard of anterior bone graft fusion was used for the fusion evaluation on CT [17]. For instance, Grade I (apparent fusion) is defined as visible bone trabeculae formation and penetration of the endplates of the upper and lower vertebral bodies; Grade II, (possible fusion) as defined by an intact bone graft and no transparent band around the head or tail, however, it was not completely remolded and integrated into the upper and lower vertebral bodies; Grade III (possibly failed fusion) as defined as an intact bone graft and the presence of a transparent band at the head or tail; Grade IV (failed fusion) defined as the bone graft being absorbed or the subsidence of the fusion cage; Grade V (unable evaluation) characteristic of cases where bone graft displacement was apparent.

Postoperative complications included C5 nerve root paralysis, cerebrospinal fluid leakage, axial pain, dysphagia, and other complications. The postoperative dysphagia was evaluated according to Bazaz criteria i.e., 0: no dysphagia, 1: mild or rare dysphagia, 2: moderate or occasional dysphagia, and 3: severe or frequent dysphagia [18].

### Statistical analysis

Data analysis was performed using the SPSS 20.0 statistical software. The Kolmogorov–Smirnov test was used to identify the normality, and all data conformed to the normal distribution. Measured data were expressed as x ± s and the independent sample t-test was used to compare two groups and in groups. The count data were compared by × 2 test between groups. P values < 0.05 were considered statistically significant.

## Results

Using the inclusion/exclusion criteria detailed above, 78 patients were retrospectively selected for this study. Based on the surgical method used, the patients were divided into 42 cases in the ACDF group (25 cases with 3 segments and 17 cases with 4 segments, a total of 143 segments) and 36 cases in the LAMP group (20 cases with 3 segments and 16 cases with 4 segments, a total of 124 segments) (Table 1).

**Table 1:** Sample characteristics of Groups A and B

Item	Group A	Group B	P-value
Age (year)	57.9 ± 8.9	62.1 ± 9.2	0.215
Gender (n)			
Male	29	25	
Female	13	11	
Follow-up time (months)	41.2 ± 3.2	40.9 ± 2.8	0.767
Operation time (minutes)	127.5 ± 30.0	131.2 ± 29.4	0.534
Blood loss (mL)	50.5 ± 23.8	326.4 ± 60.4	0.015*
Remove the drainage tube (days)	2 ( 1–2)	5 (3–7)	0.026*
hospital stay (days)	4 ( 3–7)	7 ( 5–14)	0.031*
Surgical incision (cm)			
Three-level operation	2.5 ± 0.4	10 ± 2.6	< 0.01*
Four-level operation	3.4 ± 0.5	13 ± 2.2	< 0.01*
Complications			
Axial pain	0 (0%)	3 (8.33%)	
C5 palsy	0 (0%)	2 (5.56%)	
Transient dysphagia	0 (0%)	0 (0%)	
CSF leakage	0 (0%)	1 (2.78%)	

CSF: cerebrospinal fluid. *P < 0.05 versus Group B using t-test

No significant difference in gender, age, JOA, VAS score of the upper limb, NDI score, cervical intervertebral height, functional segment height, lordosis angle, hyperextension and hyperflexion ROM, cervical ROM, and the surgical segments was observed between the two groups. The blood loss of the LAMP group was significantly higher than that of the ACDF group (P < 0.05) (Table 1). The surgical incision and hospital stay of the ACDF group were significantly shorter than that of the LAMP group (P < 0.05). There was no significant difference in the operative time between the two groups. The drainage tube removal time (median) was 2 (range 1–2) days after operation in the ACDF group and 5 (range 3–7) days after operation in the LAMP group (Table 1).

### Clinical efficacy evaluation

The postoperative JOA score was significantly improved in both groups (P < 0.05). Additionally, there was no significant difference in the improvement rate of the JOA score between the two groups. The postoperative VAS scores of the upper limb in the two groups were significantly dropped (P < 0.05). There was no significant difference in the improvement rate as measured by the NDI score between the two groups (Table 2).

**Table 2:** NDI, JOA Scores and VAS of the upper limb of Groups A and B

	Group	Preoperation	2 years after operation	P-value
NDI score	Group A	59.3 ± 5.7	59.3 ± 5.7	0.784
	Group B	59.3 ± 5.7	29.8 ± 10.1	0.854
	P value	0.814	0.758	
JOA score	Group A	10.2 ± 3.6	14.6 ± 1.2	0.048*
	Group B	9.7 ± 4.2	14.2 ± 1.7	0.035*
	P value	0.757	0.325	
VAS of the upper limb	Group A	5(3–8)	2(2–3)	0.026*
	Group B	5(3–7)	2(2–3)	0.037*
	P value	0.685	0.568	

*NDI* the neek disability index, *JOA* Japanese Orthopaedic Association, *VAS* visual analogue scale

*P < 0.05 versus Group B using t-test

All patients were evaluated according to the clinical efficacy of Odom 2 years following surgery. In the ACDF group, 32 cases were excellent, and 10 cases were good, with an excellent rate of 100%. In the LAMP group, 28 cases were excellent, and 8 cases were good, with an excellent rate of 100%. There were no significant differences in ROM in all directions and ROM loss rate between the two groups (Table 6).

### Imaging assessment

The postoperative intervertebral height and functional segment height of the ACDF group were significantly higher than those before the operation (P < 0.05) (Table 3). Additionally, the postoperative functional segment height of the ACDF group was significantly higher than that of the LAMP group (P < 0.05) (Table 4). The cervical lordosis increased significantly in the ACDF group (P < 0.05) and decreased in the LAMP group following corrective surgery. The postoperative cervical lordosis of the ACDF group was significantly higher than that of the LAMP group (P < 0.05) (Table 5). The ROM of cervical hyperextension and hyperflexion decreased in both groups, but there was no statistical difference (P > 0.05) (Table 6). At 6 months post-operation, 82 cases with Grade I fusion and 60 cases with Grade II fusion were observed in the ACDF group (Table 3). One case experienced Grade IV fusion cage sinking; the fusion rate was determined to be 99.3% (142/143).

**Table 3:** Average intervertebral height of patients with anterior cervical disorder

	Preoperative	Immediately after operation	2 years after operation	P-value
Average intervertebral height (mm)	3.91 ± 0.52	4.84 ± 0.62	4.75 ± 0.65	0.857

^*^P < 0.05 versus postoperative using t-test

**Table 4:** The total anterior length of fusion segments in Groups A and B

		Preoperative	Postoperative	P-value
Total anterior length of 3 fusion segments (mm)	Group A	58.7 ± 3.2	62.3 ± 2.9	0.048*
	Group B	57.4 ± 3.1	57.3 ± 3.0	0.957
	P value	0.752	0.035	
Total anterior length of 4 fusion segments (mm)	Group A	74.6 ± 4.1	80.7 ± 3.5	0.037*
	Group B	73.8 ± 3.6	73.6 ± 3.2	0.986
	P value	0.685	0.357	

^*^P < 0.05 versus Group B using t-test

**Table 5:** C2–7 Cobb angle of Groups A and B

	Group	Preoperative	Postoperative	C2–7 Cobb angle improvement	P-value
C2–7 Cobb angle/cervical lordosis (°)	Group A	8.7 ± 4.2	22.5 ± 6.9	13.8 ± 3.6	< 0.01*
	Group B	11.4 ± 5.4	11.2 ± 5.6	− 0.2 ± 4.7	0.958
	P value	0.758	0.258	0.178	

^*^P < 0.05 versus Group B using t-test

**Table 6:** Cervical range-of-motion of Groups A and B

	Group	Preoperative	Postoperative	P value	loss ratio
Flexion	Group A	40.63 ± 4.79	31.25 ± 4.28	0.134	19.65 ± 6.67
	Group B	42.78 ± 5.58	36.85 ± 6.64	0.239	14.62 ± 7.95
	P value	0.862	0.357		0.246
Extension	Group A	38.75 ± 4.65	32.54 ± 7.38	0.147	15.85 ± 8.69
	Group B	39.82 ± 5.85	34.38 ± 6.25	0.132	12.89 ± 4.65
	P value	0.785	0.845		0.345
Lateral bending (L)	Group A	39.69 ± 4.27	31.85 ± 4.41	0.067	17.59 ± 6.54
	Group B	45.65 ± 6.45	37.45 ± 6.68	0.075	17.78 ± 5.36
	P value	0.357	0.247		0.825
Lateral bending (R)	Group A	41.37 ± 7.85	37.85 ± 5.56	0.127	9.75 ± 3.56
	Group B	43.82 ± 6.35	38.15 ± 6.15	0.097	11.54 ± 4.52
	P value	0.687	0.728		0.358
Axial rotation (L)	Group A	72.50 ± 6.58	55.31 ± 8.65	0.125	24.58 ± 6.74
	Group B	77.24 ± 8.41	60.96 ± 9.82	0.238	22.68 ± 7.54
	P value	0.278	0.375		0.534
Axial rotation (R)	Group A	70.59 ± 7.52	53.95 ± 9.54	0.234	24.28 ± 6.15
	Group B	75.65 ± 8.40	61.25 ± 6.96	0.127	18.36 ± 7.96
	P value	0.251	0.287		0.081
Hyperextension and hyperflexion Cervical ROM	Group A	41.5 ± 11.6	26.3 ± 7.6	0.015*	36.58 ± 5.34
	Group B	43.6 ± 12.4	36.2 ± 8.5	0.037*	16.27 ± 4.78
	P value	0.571	0.125		0.021*

*ROM* range of motion

*P < 0.05 versus Group B using t-test

In the group of LAMP, three patients with axial pain complained of pain from the neck to the periscapular or shoulder area; however, the symptoms were relieved with non-steroidal anti-inflammatory drugs. The primary manifestation of C5 paralysis was deltoid weakness. After conservative treatment, the muscle strength of 2 patients returned to normal within 3 months. One case with cerebrospinal fluid leakage healed following 2 weeks of bed rest. In ACDF group, there was no case of dysphagia following operation.

## Discussion

Currently, long-level CS treatment is controversial [19]. Several studies have compared the efficacy of anterior decompression and posterior decompression for the treatment of long-level CS; however, no consensus as to the best treatment strategy has been reached. ACDF is the most common surgical intervention for CS, especially for treating single-segment CS [20]. ACDF can directly remove compression on the ventral side and open the intervertebral space, effectively restoring cervical lordosis, enlarging the intervertebral foramen, and reconstructing the stability of the degenerative segments. Treatment strategies such as the use of fusion cage and titanium plate fixation systems have been widely used in conventional ACDF [3]. Clinically, ACDF has been used to treat 1–2 segments of CS and is considered a safe and effective surgical method for cervical degenerative diseases [21]. However, the posterior open-door can enlarge the practical volume of the spinal canal to decompress the spinal cord. Although the pathology due to compression is not eliminated, compression on the spinal cord can be indirectly reduced. This is because the spinal cord drifts backward like a bowstring after the decompression [8]. Improving patients' neurologic symptoms is the ultimate goal of the operation, regardless of the operation method utilized [22]. Previous clinical studies have shown that both surgical methods can effectively alleviate neurologic symptoms [23]. In agreement with these data, we observed no significant differences between the two surgical methods in regards to their ability of alleviating neurologic symptoms. However, ACDF could be performed level by level through mini-incision resulting from the elasticity of neck skin and enough space between visceral sheath and cervical spine, and nearly no damage to paravertebral muscle occurred. In the ACDF group, the incision length was about 3 cm, the bleeding was about 50 ml, the drainage tube was removed 1–2 days after surgery, and the hospital stay was about 4 days, while the LAMP incision length was about 10 cm, the average bleeding was over 300 ml, and the drainage tube was removed about 5 days after surgery, and the hospital stay was about 7 days. The damage in mini-incision ACDF group was significantly less than that in LAMP group, and the recovery after ACDF was faster.

Consistent with previous studies, we found that cervical lordosis (C2–7) in the ACDF group increased significantly after the operation. Furthermore, the cobb angle of C2–7 in the LAMP group did not change significantly after the operation [24]. ACDF has the advantage of reconstructing cervical lordosis. It can not only restore normal physiological curvature but also restore the height of fused segments. Although ACDF is superior to LAMP in reconstructing cervical lordosis, ACDF limits C2–7 movement to some extent. Wu et al. reported that the ROM of cervical flexion and extension decreased by 28.2% and that the range of cervical rotation decreased by 14.1% after 3-level or 4-level ACDF treatment. [25] In this study, the ROM of cervical lordosis hyperextension and hyperflexion was decreased by approximately 36% in the ACDF group. Theoretically, LAMP does not affect the movement of the cervical spine. However, lamina self-fusion, muscle degeneration, and the facet joint may be essential factors contributing to reduced ROM of cervical lordosis hyperextension and hyperflexion after LAMP treatment. The ROM loss of cervical lordosis hyperextension and hyperflexion after LAMP is common in clinical studies (47.3%) [26, 27]. In this study, the ROM loss rate of cervical lordosis hyperextension and hyperflexion in the ACDF group was higher than that in the LAMP group. However, there was no significant difference in the ROM loss rate in all directions of the cervical spine after the operation between the ACDF and the LAMP groups. There was no significant difference in ROM in all directions of the cervical spine between before and after the operation in each group. This may be because the atlantooccipital and atlantoaxial joints play a significant role in flexion, extension, lateral flexion, and axial rotation [15]. Hence, it can be concluded that 3–4-level ACDF and LAMP have a negligible effect on the ROM of the cervical spine. Previous studies suggest that the increased decompression and fusion segments contribute to the increased probability of postoperative non-fusion and pseudo-joint [28]. In this study, the fusion rate was 99.3% (142/143) in ACDF group, indicating that the ROI-C plug-in self-locking fusion cage obtained robust and reliable fusion in the application of 3–4 segments. This observation may be associated with the unique anatomical design of the ROI-C plug-in interbody fusion system. Further, this design could provide ample bone graft space and make close bone contact with the endplate. Additionally, the titanium plug-in that is inserted into the vertebral body through the endplate may improve stability and facilitate early fusion. One case with the subsidence of the C6/7 segment fusion cage was defined as Grade IV fusion in 4-segment ACDF. The C6/7 was at the tail end and the tension generated by the mini-incision pushed the holder rod of the fusion cage to the head end. If the cage was inserted without following the direction of the intervertebral space, it was likely to enter the lower endplate of C7 or even the vertebral body resulting in subsidence. So the cage should be inserted parallelly to the intervertebral space, and if necessary, fluoroscopy could be used to confirm the position of cage. Then, the self-locking plug-in was knocked in when the cage was holded at the best position, which could avoid the subsidence of the fusion cage.

Both ACDF and LAMP are associated with several possible complications. Of such complications, axial pain is typical, especially in LAMP. In the current study, 42 patients in the ACDF group did not experience axial pain and 3 patients in the LAMP group reported posterior axial pain. It is understood that ligament injury of the posterior cervical muscle caused by LAMP is the main cause of potentially chronic axial pain [29–31]. Chiba et al.[32] reported that up to 28% of patients treated with LAMP experienced axial pain 14 years after the operation. However, the anterior approach caused less muscle damage. Furthermore, these patients were less likely to develop axial pain and recovered faster after the operation.

Anterior cervical surgery usually causes dysphagia and soft tissue injury. Currently, it is considered that dysphagia may be related to increased esophageal pressure during plate implantation or postoperative soft-tissue edema [33], esophageal injury, postoperative hematoma, and the formation of adhesion around the implanted cervical plate [18, 34], Several experts also indicate that dysphagia is related to the thickness of the titanium plate at the fusion level [35]. And, sometimes, it seems to be related to the long anterior cervical plate [36]. ROI-C plug-in interbody fusion cage can reduce the compression of prevertebral soft tissue. In this study, no patients developed dysphagia following ACDF treatment. No profile higher than the surface of the vertebral body after the ROI-C plug-in fusion cage was completely inserted into the intervertebral space, could avoid contact between the implant and the esophagus or other soft tissues in front of the cervical spine. This separation could eliminate any mechanical stimulation of implantation against esophagus as well as potentially avoid dysphagia. From these observations, the ROI-C plug-in fusion cage is considered a safe and effective treatment for long-level cervical disc herniation. What’s more, traditional 1–2 segments ACDF was performed small incisions (about 3 cm), but 3–4 segments ACDF was usually used long anterior cervical plate and the incisions will be extended to about 6 cm. In this article, we used ROI-C plug-in interbody fusion cage instead of long anterior cervical plate. We performed decompression and fusion of each segment in turn from top to bottom, which only required the space between the vertebral nails of Caspar inserted into the adjacent vertebrae, and the skin of the neck is elastic, so the incision (2–3 cm) was shorter than traditional ACDF. That’s why we call it “ACDF through mini-incision”.

C5 paralysis is a common complication of cervical surgery. Shou et al. [37] conducted a meta-analysis of available clinical data and found that the incidence of C5 paralysis in the ACDF and LAMP groups was 3.3% and 5.1%, respectively. In this study, the incidence of C5 paralysis in the LAMP group was 5.56%. The increased incidence may be attributed to the amplification of the spinal canal which in turn facilitates the drifting of the spinal cord to the dorsal side; thus, involving the C5 nerve root after the cervical posterior open-door surgery. The ACDF patients did not develop C5 paralysis, which may be explained by the fact that neurologic decompression in ACDF was limited to the intervertebral space, conventional enlargement of the bilateral intervertebral foramen and no overdistraction of intervertebral space. To avoid C5 paralysis, Tsuji et al. [38] limited the lamina opening angle to 53.5°to minimize spinal cord drift. Katsumi et al. [39] reduced the incidence of C5 paralysis by preventive decompression of the bilateral C4–5 intervertebral foramen. Large intervertebral height, over correction of cervical curvature, and a narrow C4–5 intervertebral foramen may be risk factors for C5 paralysis following ACDF treatment. Therefore, a reasonable plan of cervical sagittal reconstruction before the operation may reduce the incidence of C5 paralysis following ACDF treatment.

There was a limitation in this research. Because this research was a retrospective study without randomization, there might be some selection bias in this article.

## Conclusions

Both ACDF through mini-incision and LAMP are effective treatments of long-level CS. However, ACDF through mini-incision shows minor trauma, less bleeding, fast recovery and it is beneficial for cervical lordosis reconstruction.

## Data Availability

The datasets generated during and analyzed during the current study are not publicly available due to privacy and ethical restrictions but are available from the corresponding author on reasonable request.
